# Some Annual Meetings

**Published:** 1918-04-06

**Authors:** 


### SOME ANNUAL MEETINGS.

### Middlesex Hospital.

Major-General Lord Cheylesmore, who occupied the chair at the Court of Governors of the Middlesex Hospital held last week, reviewed briefly the work of the institution during 1917. Referring to the loss which the hospital had sustained by the death of Prince Christian and Mr. Arthur James, both Vice-Presidents, he mentioned the passing away of the late Lady Superintendent, Miss G. M. Thorold, after many years of faithful service, at the age of seventy-eight. After the death of her sister, she left £2,000 to the hospital, in which she always took a deep interest. During the war the hospital had cared for 8,281 wounded sailors and soldiers, 6,950 having been treated at the institution's Clacton Home, which was now entirely devoted to naval and military requirements. There had been a considerable falling-off in legacies, the amount received last year being only £4,000, as compared with £15,000 during 1916. Some idea of the increased cost of maintaining the hospital could be gathered from the fact that the price of meat had risen 150 per cent., bread 110 per cent., and milk 115 per cent., which represented an increase of no less than 14s. 3d. per bed per week over pre-war days.

### Royal Free Hospital.

At the annual meeting, held last week, special mention was made of the Medical School for Women, which suffered a great loss last December by the death of its President, Dr. Garrett Anderson, who was its moving and guiding spirit and had raised £35,000 to build and equip a new school on the site of the old one. The school's hospitality continues to be offered to five women medical students of Allied nationality. Dr. Scharlieb spoke of the great services that men and women now being trained in medical schools could render to prevent infantile mortality. Over £16,000 has been raised towards the fund for the future extension of the hospital, and, although no definite action can be taken in that direction at present, it is hoped shortly to relieve congestion by converting a portion of the existing nurses' quarters into accommodation for twenty patients, the nurses being provided for elsewhere.

### University College Hospital.

Like many other similar institutions, the administrative work of which has been materially assisted owing to the fact that women have been given seats upon the Boards of Management, the University College Hospital has added to the list of its General Committee the names of two well-known hospital supporters—Princess Marie Louise and Lady Owen Phillips. In making this announcement at the meeting held last week, Sir Ernest Hatch, who presided, gave it as his opinion that in the near future no hospital Board would be considered complete unless it comprised members of both sexes. The Duke of Bedford was elected President of the hospital for the ensuing year. During 1917 a large number of wounded sailors and soldiers had been cared for, and a sum of £15,425 had been received from the Government towards the cost of so doing. The ordinary expenditure of the institution, which amounted to over £49,000, slightly exceeded the income and showed an increase of 16 per cent. on the sum expended in 1916. Twenty-eight extra beds had been provided. Out-patients to the number of 5,416, whose attendances totalled 131,746, had been treated.

### London Temperance Hospital.

At the annual Court of Governors of the London Temperance Hospital, Hampstead Road, held last week, the Bishops of Willesden and Croydon strongly denounced alcohol as the root of much evil, and pointed to the great work which the hospital was doing as proof that the treatment of diseases could be accomplished without it. Since the establishment of the institution forty-five years ago, whereas 40,313 in-patients had been treated and 34,804 of them either cured or relieved, in only 113 cases had alcohol been administered. The Chairman intimated that during the past year 189 soldiers had been admitted to the hospital, making a total of 589 cared for during the war. Mrs. Tinson White has been appointed as salaried supervisor of massage treatment, with Miss Blair as her assistant.

### National Hospital for the Paralysed and Epileptic.

At the annual meeting of the Governors of the National Hospital for the Paralysed and Epileptic, held last week at Queen Square, W.C., under the presidency of Sir Frederick Macmillan, Chairman of the Board of Management, reference was made to the increasing services which the institution was rendering to sailors and soldiers. (See under Institutional News, p. 3.) The hospital's income has increased, but the problem of finding £27,000, which represents approximately the expenditure last year for what before the war cost £18,000, is causing the Board some anxiety. Nearly a thousand in-patients and 7,432 out-patients were treated in 1917.

### East London Hospital for Children.

Much gratification was expressed at the annual meeting of Governors, held last week, at the fact that though the Board of Management was unable to get through the year without help from its bankers, owing to timely assistance by way of increased grants from various funds, including those inaugurated at the end of 1916 by the Duchess of Portland, Emily Lady Ampthill, and the proprietors of *Punch*, it was able to repay what had been borrowed and thus to start the jubilee year of the hospital free of debt. Because of the shortage of nurses and the closing of the isolation block for the greater part of the year, the number of in-patients had decreased. New out-patients were also fewer than in 1916. The expenditure last year had risen by nearly £1,000.

### Leicester Royal Infirmary.

At the 145th Anniversary Meeting of the Leicester Royal Infirmary, presided over by Sir Thomas Cope, Bart., on Wednesday, March 27, the attendance showed the interest which is taken generally in the institution.

Mr. T. Fielding Johnson, jun., J.P., the chairman of the board, in moving the adoption of the report, referred to the considerable curtailment in its pages, which he was certain would be approved of as a war measure. The work of the year had been a record in spite of record difficulties; the generosity and patriotism of the district and the continuity and increasing interest and energy of the Saturday Hospital Society, the returns of which showed an increased income of about £500, not only enabled the institution to pay its way, but the investments had been increased by approximately £10,000. This included £3,000 which was now set aside each year to be ready for future

(*Continued on p.* iv.)

